# Seed coating with plant growth-promoting rhizobacteria enhances potato (*Solanum tuberosum* L.) growth and yield

**DOI:** 10.3389/fmicb.2025.1738090

**Published:** 2026-03-06

**Authors:** Jinxue Hu, Congchao Xiang, Yao Lu, Mingfei Jia, Zhiming Feng, Shuqing Zhang

**Affiliations:** 1 Shijiazhuang Academy of Agriculture and Forestry Sciences, Shijiazhuang, China; 2 Laboratory of Potato Genetic Improvement and Germplasm Innovation in Shanxi Province, Shanxi Agricultural University, Taiyuan, China

**Keywords:** soil enzymes activity, soil nutrient, nutrient accumulation, nutrient translocation, net photosynthetic rate

## Abstract

Utilizing beneficial plant growth-promoting rhizobacteria (PGPR) offers an effective approach for achieving sustainable crop production. However, research on the application and mechanisms of PGPR seed coating in potato (*Solanum tuberosum* L.) remains limited. Therefore, we conducted a two-year field experiment involving five seed-coating treatments: untreated (CK), chemical coating (CB), *Bacillus velezensis* coating (SM), and two composite formulations, CM1 (*Bacillus subtilis* + *Paenibacillus mucilaginosus*) and CM2 (*Bacillus subtilis* + *Bacillus licheniformis*). The results showed that PGPR markedly improved soil NO₃^−^-N and available P contents by stimulating carbon (C), nitrogen (N), and phosphorus (P) cycling enzymes. During potato flowering stages, soil NO₃^−^-N and available P increased by 16.29 and 17.29%, respectively. PGPR also increased plant height and stem diameter by 10.89 and 34.46% relative to CB, and elevated SPAD values and net photosynthetic rate (*P_n_*) at flowering by 20.22 and 32.22%, respectively. At maturity, potato aboveground, root, and tuber dry matter under PGPR increasing by 31.27, 44.21, and 41.88% compared with CB. Enhanced root biomass and nutrient acquisition promoted nutrient redistribution in potato, increasing N and P translocation to tubers by 17.13 and 50.48%, respectively. CM2 exhibited the highest tuber N and P accumulation, increasing by 66.74 and 55.25%, and achieved a 38.9% higher yield compared with the other treatments. Overall, PGPR enhanced soil nutrient availability, plant photosynthetic performance, nutrient acquisition, and nutrient translocation, thereby supporting greater biomass accumulation and promoting sustainable potato production. The PGPR seed coating represents an effective and scalable strategy for achieving resource-efficient and sustainable potato production.

## Highlights

- PGPR seed coating improved soil nutrient availability by stimulating nutrients cycling enzymes.
- PGPR strengthened root system development and enhanced chlorophyll synthesis.
- PGPR enhanced N and P uptake and facilitated translocation to tubers.
- PGPR-based seed coating increased potato biomass and yield.

## 1 Introduction

As the world’s fourth major staple crop, potato (*Solanum tuberosum* L.) possesses remarkable yield potential and rich nutritional content, making it vital for ensuring global food security (Alkhaled and Wang, 2025). More than one billion people consume potatoes globally, with total production exceeding 350 million metric tons annually (Birch et al., 2012). However, potato is recognized as a nutrient-intensive crop, typically demanding around 250 kg N ha^−1^ and 150 kg P ha^−1^ to reach its optimal yield potential (Naqqash et al., 2022). While such inputs have contributed to yield improvements, they have also raised concerns regarding soil degradation, nutrient imbalances, and disruptions to crop physiological processes (Wang et al., 2025; Lu et al., 2025; Bailey-Serres et al., 2019; Dobermann et al., 2022). In addition to its high nutrient demand, potato production faces challenges related to seed health. As a vegetatively propagated crop, potato relies on cut seed tubers, whose exposed surfaces are highly vulnerable to soil-borne pathogens. Consequently, seed treatment is essential for preventing infection and ensuring healthy seedling establishment (Birch et al., 2012). Chemical seed treatments are widely used and can effectively suppress pathogen invasion. However, their excessive or long-term application may induce phytotoxicity, hinder sprouting and early growth, disturb soil microbial community structure, reduce microbial activity, and contribute to environmental contamination (Paulikienė et al., 2025). Overall, these challenges highlight underlying systemic constraints in potato cultivation, particularly regarding seed health, microbial stability, and early plant development, which are not sufficiently addressed by current practices. Identifying biologically based seed-treatment solutions that enhance rhizosphere functioning and crop establishment has therefore become increasingly important.

In agriculture, plant microbiota is considered the cornerstone of the next green revolution, as it can improve crop performance (Finkel et al., 2020). Among them, plant growth-promoting rhizobacteria (PGPR) are especially important. These beneficial bacteria colonize the rhizosphere and sometimes penetrate root tissues, promoting plant growth and improving plant performance (Vocciante et al., 2022; Mawar et al., 2023). PGPR can influence plant nutrition by modifying nutrient uptake efficiency and regulating the rate of plant growth and development (Etesami and Adl, 2020; Jalal et al., 2023). In terms of nutrient supply, PGPR can significantly improve the uptake and utilization efficiency of essential elements such as nitrogen, phosphorus via nitrogen fixation, phosphate solubilization, and potassium solubilization (Elhaissoufi et al., 2022; Khourchi et al., 2023; Kour et al., 2019; Naqqash et al., 2016; Zak et al., 2017). For instance, some PGPR are capable of fixing atmospheric nitrogen into ammonium, a form readily available for plant uptake, thus decreasing dependence on synthetic nitrogen fertilizers (Sun et al., 2020). Others secrete organic acids and phosphatases to dissolve insoluble phosphate compounds in the soil, increasing the content of available phosphorus (Chea et al., 2024); they can also produce organic acids, chelating agents, or potassium-solubilizing enzymes to release mineral potassium into soluble forms, markedly enhancing the supply of available potassium (Chen et al., 2022). Seed coating with PGPR can promote the early establishment of beneficial plant-microbe associations in the potato rhizosphere, thereby enhancing root development and nutrient acquisition. Selecting suitable PGPR strains is therefore essential to maximize their growth-promoting effects.

PGPR can not only directly enhance crop nutrient uptake by stimulating root activity but also increase the availability of soil nutrients. According to Ren et al. (2021), the incorporation of PGPR significantly increased soil total nitrogen by 100% and total potassium by 22%. This improvement is mainly attributed to the enhanced root growth and rhizosphere enzyme activities that promote nutrient mineralization and the transformation of nutrients into plant-available forms (Shang et al., 2023). PGPR can secret or induce a variety of key soil enzymes, including urease, acid phosphatase, alkaline phosphatase, sucrase, and peroxidase (Bigatton et al., 2024). Enzymatic activities drive the degradation and biochemical turnover of soil organic substrates, promoting the liberation and circulation of nutrients such as nitrogen, phosphorus, and potassium, which ultimately enhance nutrient supply in the rhizosphere environment (Liu et al., 2021; Wei et al., 2020; Yan et al., 2024). Although the functions of PGPR in improving nutrient supply, enhancing soil biological activity (Kour et al., 2019; Naqqash et al., 2016; Yan et al., 2024), and promoting crop growth (Imran et al., 2024) have been extensively demonstrated, their application in potato (*Solanum tuberosum* L.) under field conditions remains relatively limited. In addition, compared with single-strain seed coating, composite PGPR formulations combine bacterial species with complementary functional attributes-including nitrogen fixation, phosphate and potassium solubilization, phytohormone production, and stimulation of soil enzyme activities-which may generate synergistic effects on rhizosphere biochemical functioning and nutrient acquisition. Such multi-strain combinations are expected to provide more stable and broader-spectrum benefits than individual strains. However, comprehensive field evaluations of composite PGPR seed-coating strategies in potato remain limited, especially regarding their integrated effects on soil nutrient dynamics, nutrient accumulation and translocation, photosynthetic performance, and yield formation. This knowledge gap underscores the need to investigate whether composite PGPR offer superior advantages over single-strain coatings under real production conditions.

This two-year field investigation aimed to clarify how plant growth-promoting rhizobacteria (PGPR) stimulate potato growth by integrating soil biochemical dynamics with plant physiological traits. We hypothesized that (1) PGPR seed coating would enhance potato growth, nutrient accumulation, and yield formation; (2) these responses are likely linked to enhanced soil nutrient accessibility and the stimulation of crucial enzymes responsible for C, N and P cycling; and (3) PGPR would strengthen soil–plant interactions, thereby enhancing nutrient uptake and translocation efficiency and promoting sustainable potato productivity.

## 2 Materials and methods

### 2.1 Experimental site and weather conditions

**Figure 1:**
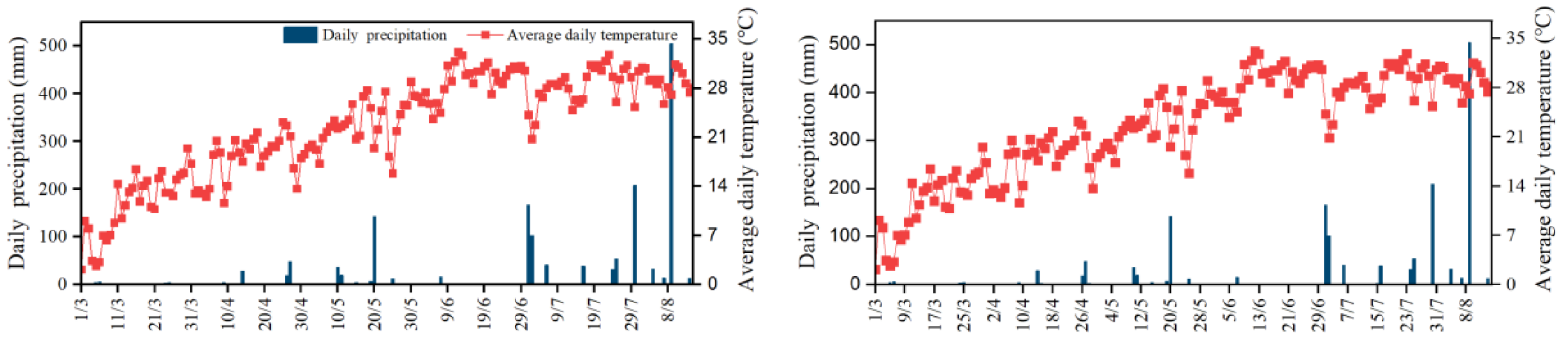
Rainfall and temperature in potato growing season in 2023–2024.

The field experiment took place between 2023 and 2024 in Zhaoxian County, Shijiazhuang, Hebei Province, China (37°45′N, 114°46′E). The site lies in the south-central region of the North China Plain and is characterized by a warm temperate, semi-humid monsoon climate of continental type. The elevation is about 45 m above sea level, having an average temperature of 13.5–14.0 °C, precipitation of 450–600 mm each year, and a frost-free span of around 190–210 days. The predominant soil types is light loam fluid-brown soil, with medium-to-low organic matter content in the plow layer. The local cropping system is a double-cropping rotation per year. The field followed a potato-maize double-cropping rotation. Potato was planted in early March and harvested in late June. Maize was then sown in early July and harvested in early October. Before potato planting, the soil was moldboard-plowed and ridged, and crop residues from the previous season were incorporated. Standard fertilization and irrigation practices were applied for each crop according to local agronomic recommendations. Table 1 summarizes the main physicochemical features of soils at the experimental site, and Figure 1 shows the precipitation and temperature patterns during the potato growing seasons in 2023 and 2024.

**Table 1:** Nutrient status of farmland soil experimental field.

Soil depth	Alkali-hydrolyzable N (mg/kg)	Available phosphorus (mg/kg)	Available potassium (mg/kg)	Organic matter (g/kg)	pH value
0–20 cm	126.5	26.8	148.6	21.56	8.09

### 2.2 Experimental design and treatments

The potato cultivar “Shishu No. 10” used in this study was supplied by the Shijiazhuang Academy of Agricultural and Forestry Sciences. The field experiment followed a randomized block design with three replicates and comprised five seed treatment modes: untreated control (CK), chemical coating (CB), coating with *Bacillus velezensis* (2.0 × 10^8^ CFU g^−1^, SM), coating with *Bacillus subtilis* (2.0 × 10^8^ CFU g^−1^) combined with *Paenibacillus mucilaginosus* (2.0 × 10^8^ CFU g^−1^, CM1), and coating with *Bacillus subtilis* (2.0 × 10^8^ CFU g^−1^) combined with *Bacillus licheniformis* (3.0 × 10^8^ CFU g^−1^, CM2). *B. velezensis*, a functionally versatile PGPR species, has been widely reported to enhance potato growth and improve tolerance to environmental stresses through both direct and indirect mechanisms (Lu et al., 2025). *B. subtilis* is a widely applied PGPR species capable of promoting root growth, inducing systemic resistance, and enhancing nutrient uptake efficiency (Bailey-Serres et al., 2019). *P. mucilaginosus* is an effective mineral-solubilizing bacterium that releases potassium and phosphorus from soil minerals and improves nutrient availability (Dobermann et al., 2022). *B. licheniformis* contributes to plant growth through phosphate solubilization, phytohormone production, and enhancement of plant stress tolerance (Trivedi et al., 2020). The complementary functional traits of these strains justify their use in composite formulations (CM1 and CM2) to achieve synergistic effects on rhizosphere functioning and crop performance. The size of each plot was 25 m long and 5 m wide. All microbial agents were applied in powder formulation. The specific methods are shown in Table 2.

**Table 2:** Application rates of different types of methods.

Treatment	Processing method	Chemical agent	Microbial agent
CK	Unprocessed	0 kg ha^−1^	0 kg ha^−1^
CB	Chemical biocide seed coating	1.5 kg ha^−1^ thiophanate methyl +0.6 kg ha^−1^ Kasumin +45 kg ha^−1^ talcum powder	0 kg ha^−1^
SM	Single microbial seed coating	0 kg ha^−1^	50 kg ha^−1^
CM1	Composite microbial seed coating 1	0 kg ha^−1^	22.5 kg ha^−1^
CM2	Composite microbial seed coating 2	0 kg ha^−1^	1.5 kg ha^−1^ + 45 kg ha^−1^ talcum powder

Certified seed tubers were supplied by the Shijiazhuang Academy of Agriculture and Forestry Sciences. Before planting, the tubers were cut into pieces and treated with different seed-coating formulations as described in Table 2. After coating, the tuber pieces were maintained at room temperature (25 °C) for 20 days to ensure uniform sprouting. Potatoes were planted using a single-ridge planting pattern, with an in-row spacing of 20 cm and a row spacing of 75 cm, resulting in a planting density of approximately 67,500 plants ha^−1^. A compound fertilizer (N:P:K = 15:15:15) was applied as the basal fertilizer prior to planting. Drip irrigation was supplied according to local agronomic recommendations, with irrigation events scheduled every 7–10 days depending on weather conditions and soil moisture status. Pest and disease management followed standard regional practices, and fungicides or insecticides were applied only when economic thresholds were exceeded. All other field management practices were consistent with local commercial potato production.

### 2.3 Determination items and methods

#### 2.3.1 Measurement of potato agronomic traits

Sampling was conducted at the seedling, squaring, flowering, and maturity stages of potato. At each stage, three potato plants with uniform growth and free of disease were randomly selected from each plot (three replicates, nine plants per treatment in total) to measure plant height, stem diameter, photosynthetic rate, and dry matter accumulation.

For plant sampling, an intact soil monolith measuring 10 × 20 cm (width × depth) was carefully dug out to preserve the entire plant structure, and loosely adhering soil was gently shaken off. The plants were brought back to the laboratory, where they were divided into shoots, roots, and tubers and then placed in paper bags for subsequent analysis. Samples were pretreated at 105 °C for 30 min to inactivate enzymes, then dried at 80 °C until constant weight for dry matter measurement.

Measurements were conducted as follows: plant height was measured using a measuring tape by recording the vertical distance from the stem base to the apex of the highest leaf; stem diameter was determined with an electronic vernier caliper at approximately 2 cm above the soil surface on the thickest stem; SPAD was measured in the adaxial surface of leaves with a modulated chlorophyll fluorometer (Opti-Sciences, OS1p) in the pulse amplitude modulation mode (Finkel et al., 2020); the net photosynthetic rate was recorded with a LI-6400 portable photosynthesis analyzer under clear-sky conditions, calm conditions in the morning, with fully expanded, healthy leaves selected for measurement (Cordovez et al., 2019); dry matter weight was determined with an electronic balance.

#### 2.3.2 Measurement of nitrogen and phosphorus contents in potato plants

After drying, plant samples were milled and screened through a 0.5 mm mesh. Total nitrogen (TN) contents and total phosphorus (TP) contents in different plant organs (shoots, roots, and tubers) were determined after digestion with a sulfuric acid-hydrogen peroxide solution. Total N was analyzed with an automatic Kjeldahl analyzer, while total P estimation followed the vanadium-molybdenum yellow colorimetric procedure. Nitrogen and phosphorus accumulation, translocation, and their contribution rates to tubers were computed as follows (Mawar et al., 2023):


$$\begin{array}{l} \text{Plant}\;N\;(\begin{array}{l} \text{or}\;\\ P \end{array})\;\text{accumulation}\;(\begin{array}{l} \mathrm{mg}/\\ \text{plant} \end{array})=N\;(\text{or}\;P)\;\text{concentration}\;(\mathrm{mg}/g)\times \\ \mathrm{dry}\;\text{matter weight}\;(g/\text{plant}); \end{array}$$

$$\begin{array}{l} \text{Plant}\;N\;(\text{or}\;P)\;\text{translocation amount}\;(\mathrm{mg}/\text{plant})=\\ N\;(\text{or}\;P)\;\text{accumulation}\;\mathrm{at}\;\text{flowering stage}-\\ N\;(\text{or}\;P)\;\text{accumulation}\;\mathrm{at}\;\text{maturity stage}; \end{array}$$

$$\text{Plant}\;N\;(\text{or}\;P)\;\text{translocation rate}\;(\%)=[\begin{array}{l} N\;(\text{or}\;P)\;\text{translocation}\;\\ \text{amount}/\\ N\;(\text{or}\;P)\;\text{accumulation}\;\\ \mathrm{at}\;\text{flowering stage} \end{array}]\times 100\%;$$

$$\text{Plant}\;N\;(\text{or}\;P)\;\text{contribution rate}\;(\%)=[\begin{array}{l} N\;(\text{or}\;P)\;\text{translocation}\;\\ \text{amount}/\text{tuber}\;N\;(\text{or}\;P)\;\\ \text{accumulation}\;\mathrm{at}\;\\ \text{maturity stage} \end{array}]\times 100\%.$$

#### 2.3.3 Determination of soil nutrient contents and enzyme activities

Soil was sampled with an auger from the 0–20 cm layer during the seedling, squaring, flowering, and maturity stages of potato growth. Within each plot, three points were randomly chosen, and the collected soils were pooled and thoroughly homogenized to obtain one representative sample. The samples were then transported to the laboratory, gently sieved through a 100-mesh screen, and preserved at −4 °C for subsequent analyses of nutrient status and enzyme activities.

Soil ${\mathrm{NO}}_{3}^{–}$ -N and ${\mathrm{NH}}_{4}^{+}$ -N were obtained by extraction with 0.5 M K₂SO₄ solution. ${\mathrm{NO}}_{3}^{–}$ -N was measured by ultraviolet spectrophotometry, whereas ${\mathrm{NH}}_{4}^{+}$ -N was quantified by the indophenol blue colorimetric procedure (Zhao D. Q. et al., 2022). Available P was obtained by extraction with 0.5 M NaHCO₃ solution and quantified through the molybdenum blue colorimetric procedure as outlined by Olsen et al. (1954). The activities of β-glucosidase (BG), β-xylosidase (BX), cellulase (CE), N-acetyl-β-D-glucosaminidase (NAG), leucine aminopeptidase (LEU), urease (URE), and acid phosphatase (AP) were determined using a microplate reader based on the procedures outlined by Zhao et al. (2024). These enzyme activities were measured at the maturity stage, and only in the final year of the experiment. Enzyme activities with similar functions were normalized as follows: C-related enzymes (BG, BX, and CE), N-related enzymes (NAG and LEU), and P-related enzyme (AP), following the approach of Ma et al. (2022) and Zhou et al. (2023):


C−acq=(BG+BX+CE)/3

#### 2.3.4 Analysis of potato yield and economic benefits

During potato harvest, three strips (3 m x 0.75 m each) were taken from each plot (total 6.75 m^2^) for in-situ digging and tuber fresh-weight measurement. Each plot was also harvested separately and the total plot yield recorded. Each treatment was replicated three times, and the average of these replicates was used to represent the treatment yield. Plot yield was converted to a standard unit-area basis using the measured fresh weight and the actual plot area. To reduce the influence of moisture differences, sample moisture content was recorded and fresh- to dry-basis conversions were performed as needed.

*Yield conversion*


$$\text{Yield}\;(\mathrm{kg}\;{\mathrm{ha}}^{-1})=[\text{Plot fresh weight}\;(\mathrm{kg})/\text{Plot area}\;(m^{2})]\times 10,000$$

*Economic benefit analysis*

Gross value was estimated from the mean yield of each treatment and the market price, and net profit was calculated by deducting input costs.


$$\text{Gross value}\;(\text{yuan}\;{\mathrm{ha}}^{-1})=\text{Yield}\;(\mathrm{kg}\;{\mathrm{ha}}^{-1})\times \text{Market price}\;(\text{yuan}\;{\mathrm{kg}}^{-1})$$

$$\text{Total cost}\;(\text{yuan}\;{\mathrm{ha}}^{-1})=\Sigma (\begin{array}{l} \text{seed tubers}+\text{fertilizers}+\\ \text{pesticides}+\text{irrigation}+\\ \text{machinery}+\text{labor} \end{array})$$

where seed tubers: 7,500 yuan ha^−1^; fertilizers: 7,500 yuan ha^−1^; pesticides: 3,000 yuan ha^−1^; irrigation: 750 yuan ha^−1^; machinery: 9,000 yuan ha^−1^; labor: 6,000 yuan ha^−1^.


$$\mathrm{Net}\;\text{profit}\;(\text{yuan}\;{\mathrm{ha}}^{-1})=\text{Gross value}-\text{Total cost}$$

#### 2.3.5 Statistical analysis

All data analyses and figure visualization were carried out using R software (R Core Team, 2023). The Shapiro–Wilk and Levene tests were used to evaluate residual normality and variance homogeneity. Variations in the measured variables among seed-coating treatments were evaluated through one-way ANOVA, and mean separation was performed using Duncan’s multiple range test at *p* < 0.05. Associations among potato yield, nutrient accumulation and translocation, plant growth characteristics, soil attributes, and enzymatic activities were examined through Pearson correlation and Mantel tests (999 permutations) using the ggcor package in R.

## 3 Results

### 3.1 Soil N and P availability and C/N/P cycling enzyme activities

Soil NO₃^−^ and available P gradually decreased with plant growth. At the squaring and flowering stages, soil NO₃^−^ contents under PGPR (SM, CM1, and CM2) were 16.18 and 16.29% higher than CK and CB, respectively, while soil available P under PGPR was 25.10 and 17.29% higher than CK and CB, respectively. The activities of C-cycling enzymes in PGPR (SM, CM1, and CM2) were 22.41% higher than in CB. CM2 exhibited higher N-cycling enzyme activities than SM and CM1 by 13.26%, and showed increases of 5.14 and 8.37% compared with CK and CB, respectively. Combined PGPR (CM1 and CM2) significantly enhanced P-cycling enzyme activities by 11.03% relative to CB and SM, and by 20.81% compared with CK.

### 3.2 Potato growth and photosynthetic characteristics

**Figure 2:**
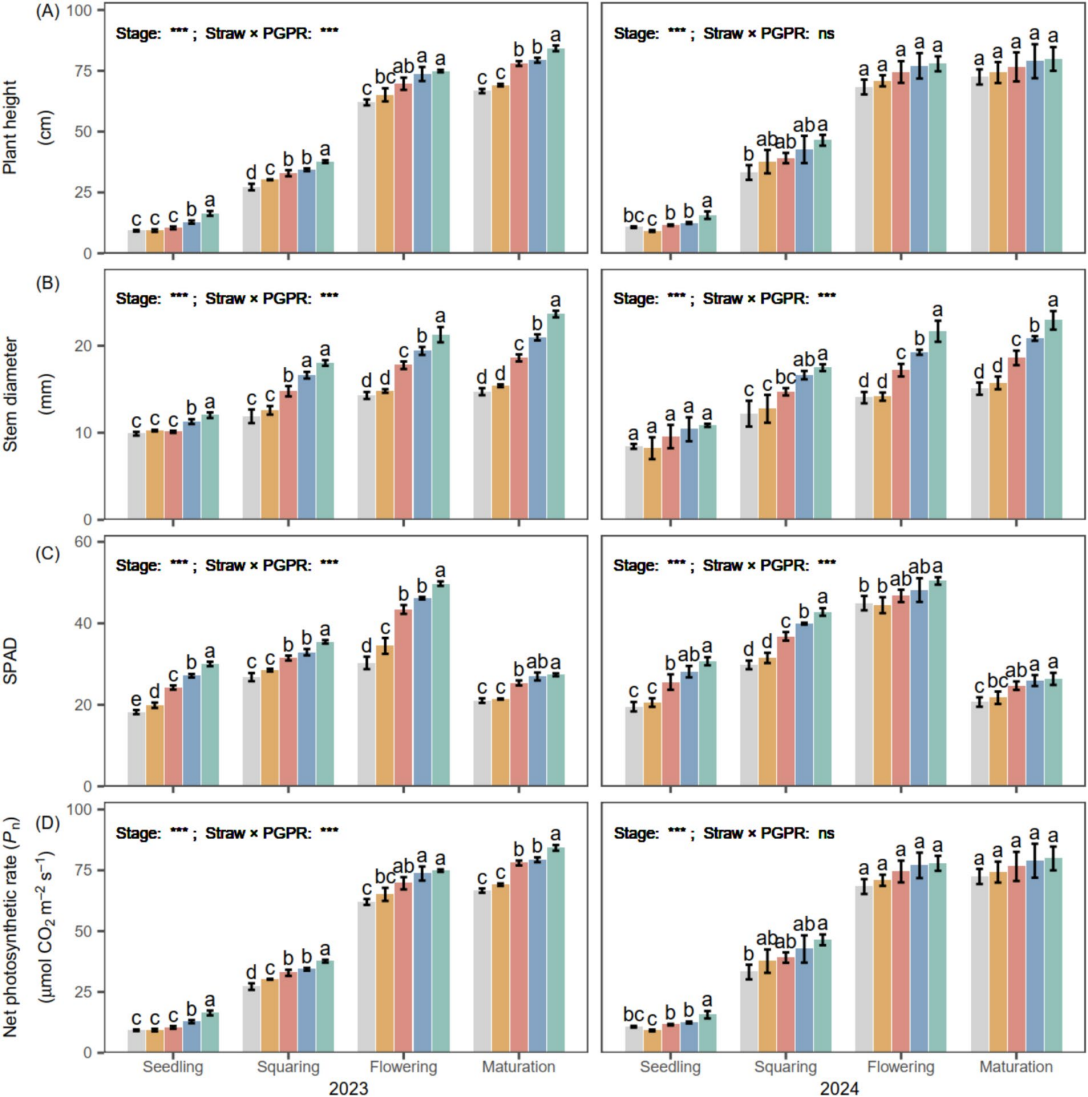
Effect of different PGPR on **(A)** plant height, **(B)** stem diameter, **(C)** SPAD chlorophyll index, and **(D)** net photosynthetic rate (*P_n_*) of *Solanum tuberosum* L. during 2023–2024. Different lowercase letters denote significant differences (*p* < 0.05) among seed-coating treatments at each potato growth stage. “Stage” and “Stage × PGPR” represent the main effects and their interaction in the two-way ANOVA (ns, not significant; *, **, ***, significant at increasing levels).

During the entire growth period of potato, both plant height and stem diameter gradually increased in both years (Figure 2), and they remained consistently higher than CK. PGPR (SM, CM1 and CM2) resulted in greater plant height and stem diameter, which were on average 10.89 and 34.46% higher than CB at maturity, respectively. Throughout the growth period, SPAD and *P_n_* peaked at the flowering stage, showing increases of 20.22 and 32.22% over CB.

### 3.3 Dry matter accumulation and partitioning

The accumulation of aboveground biomass showed a steady rise from the seedling phase to flowering, after which it reached a plateau during the maturation stage. PGPR (SM, CM1 and CM2) had significantly greater aboveground dry matter than CK and CB, showing an average increase of 31.27% at maturity across the 2 years. Root dry matter increased gradually during the entire growth period. PGPR significantly promoted root dry matter accumulation, which peaked at maturity and was on average 44.21% higher than CB. Tuber dry matter exhibited rapid accumulation from the flowering stage to maturity, reaching its peak at maturity, with an average increase of 41.88% relative to CB across the 2 years.

### 3.4 Nitrogen and phosphorus accumulation and partitioning

**Figure 3:**
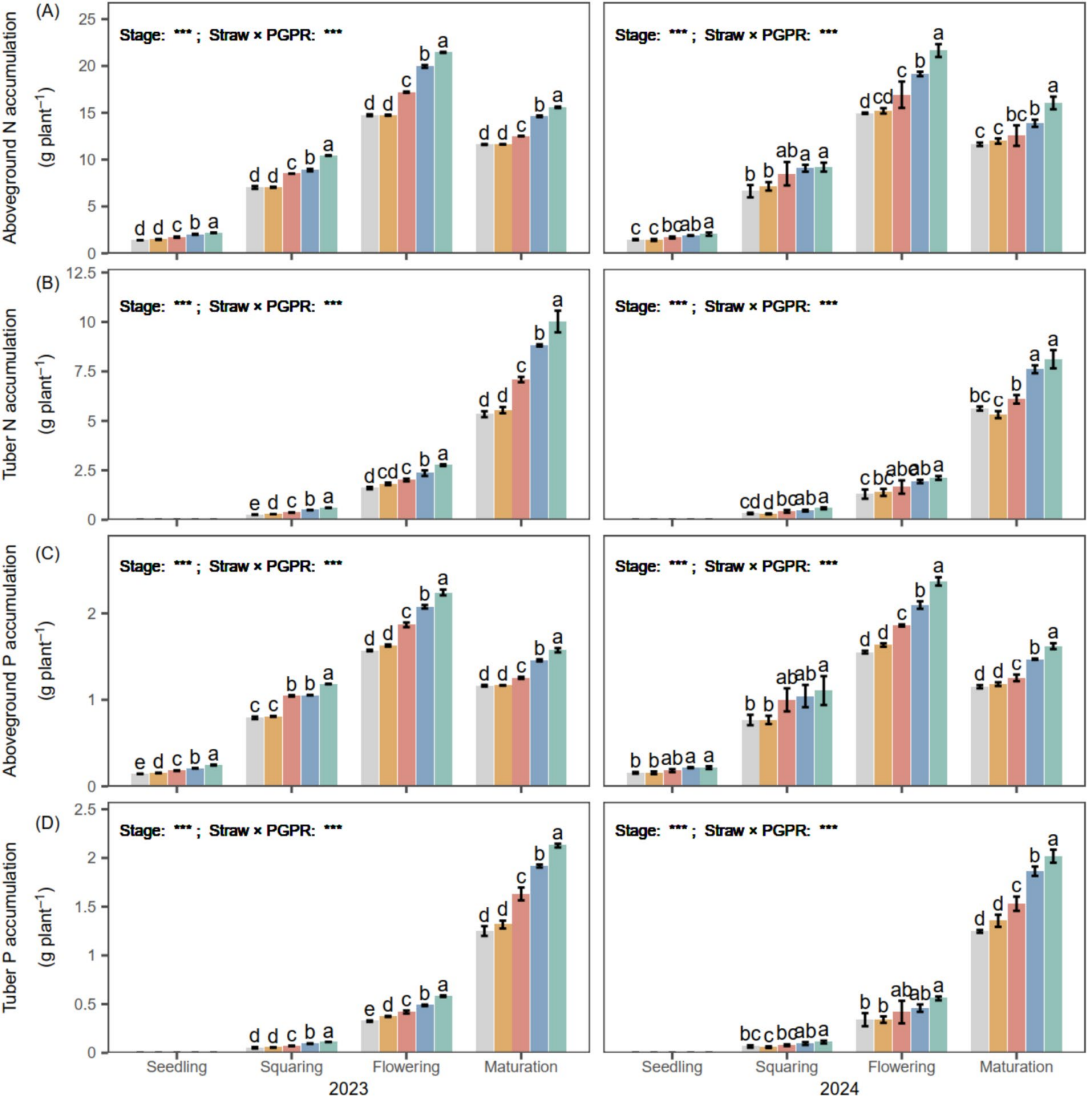
Aboveground **(A,C)** and tuber **(B,D)** nitrogen (N) and phosphorus (P) accumulation of potato under different PGPR in 2023 and 2024. Different lowercase letters denote significant differences (*p* < 0.05) among seed-coating treatments at each potato growth stage. “Stage” and “Stage × PGPR” represent the main effects and their interaction in the two-way ANOVA (ns, not significant; *, **, ***, significant at increasing levels).

Aboveground N and P accumulation increased progressively from the seedling to the flowering stage (Figure 3). PGPR (SM, CM1 and CM2) enhanced N and P accumulation relative to CK and CB. CM2 had the highest aboveground N and P accumulation, being 43.93 and 41.38% higher than CB at the flowering stage in both years. At maturity, aboveground N and P decreased compared with the flowering stage, with an average decrease of 4.39 g plant^−1^ and 0.56 g plant^−1^ over the 2 years. Tuber N and P showed rapid accumulation from the flowering stage to maturity, reaching 5.49–9.07 g plant^−1^ and 1.25–2.07 g plant^−1^ at maturity, respectively. Compared with CB, tuber N accumulation under CM1 and CM2 increased by 51.06 and 66.74%, while tuber P accumulation increased by 41.60 and 55.25%, respectively, across the 2 years.

### 3.5 Nitrogen and phosphorus translocation and remobilization characteristics

**Figure 4:**
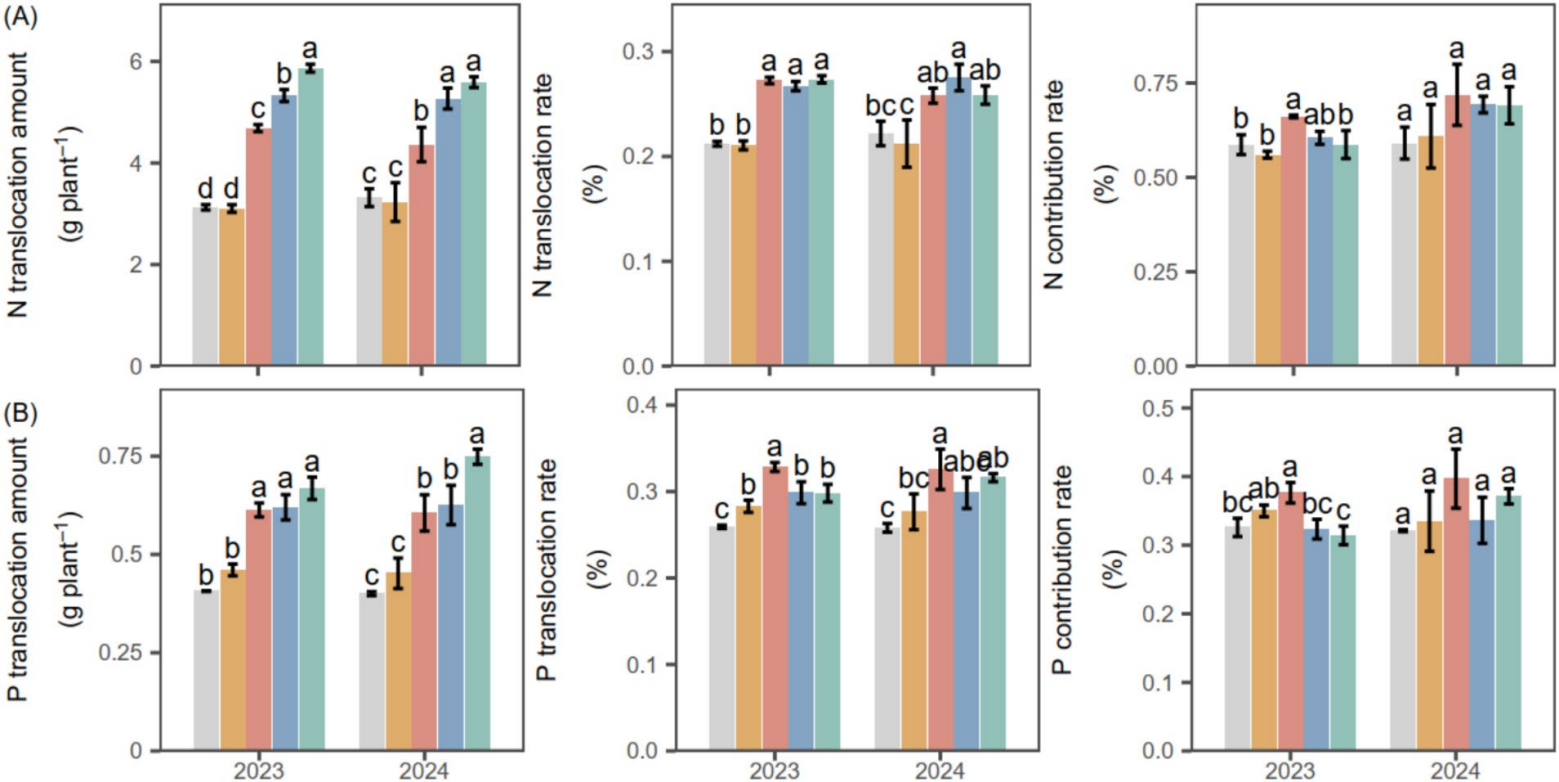
**(A, B)** Translocation amount, rate, and contribution of nitrogen (N) and phosphorus (P) in potato plants under various PGPR during 2023 and 2024. Different lowercase letters denote significant differences (*p* < 0.05) among seed-coating treatments at each potato growth stage. “Stage” and “Stage × PGPR” represent the main effects and their interaction in the two-way ANOVA (ns, not significant; *, **, ***, significant at increasing levels).

In both 2023 and 2024, only PGPR (SM, CM1 and CM2) enhanced the translocation of N and P (Figure 4). For N, combined PGPR (CM1, CM2) increased N translocation by 21.95% compared with SM and by 67.36% compared with CK, on average. PGPR (SM, CM1, and CM2) increased the N translocation rate by an average of 17.13% relative to CK and CB. In both years, the N contribution rate of SM was 7.42% higher than the combined PGPR (CM1, CM2) and 17.57% higher than CK and CB. For P, PGPR (SM, CM1, and CM2) promoted P translocation by an average of 50.48% compared with CK and CB over the 2 years. The P translocation rate under SM was 8.46% higher than the combined PGPR (CM1, CM2) and 22.00% higher than CK and CB, with statistical significance observed only in 2023. On average, the P contribution rate in SM was 3.88% higher than in the other treatments across the 2 years.

### 3.6 Yield and economic benefits

**Figure 5:**
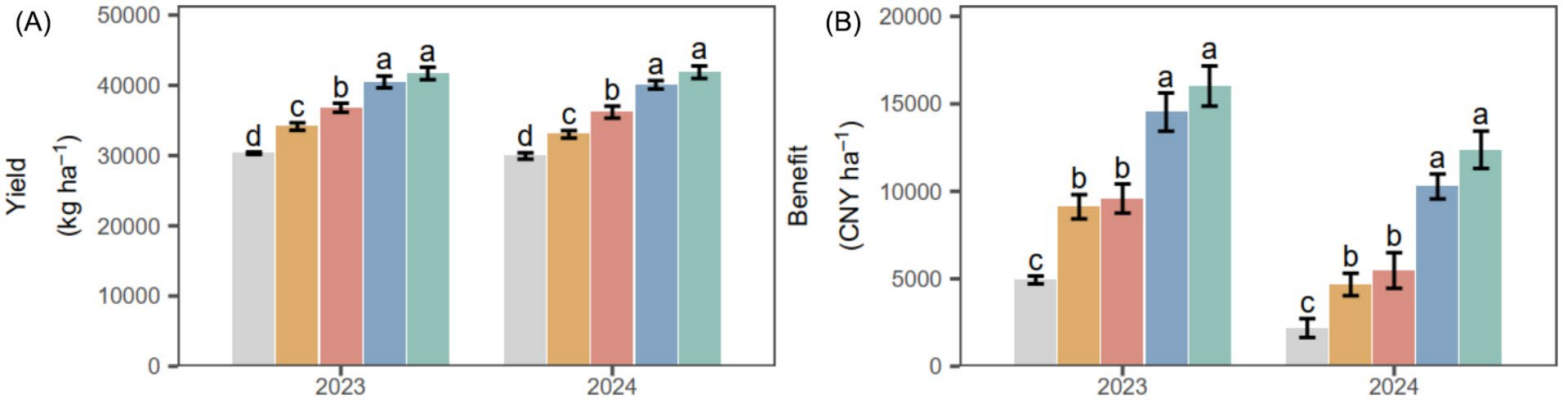
Potato yield **(A)** and economic benefit **(B)** under different PGPR in 2023 and 2024. Different lowercase letters denote significant differences (*p* < 0.05) among seed-coating treatments at each potato growth stage. “Stage” and “Stage × PGPR” represent the main effects and their interaction in the two-way ANOVA (ns, not significant; *, **, ***, significant at increasing levels).

PGPR significantly improved potato yield and economic benefits (Figure 5). The combined PGPR (CM1 and CM2) increased yield by an average of 7626.73 kg ha^−1^ (22.83%) relative to CB and SM, with an income increase of 6087.88 CNY ha^−1^ over the 2 years. Compared with CK, combined PGPR (CM1 and CM2) increased yield by 10,889.79 kg ha^−1^ (36.13%) and increased income by 9,742.84 CNY ha^−1^ on average across the 2 years.

### 3.7 Correlation analysis

**Figure 6:**
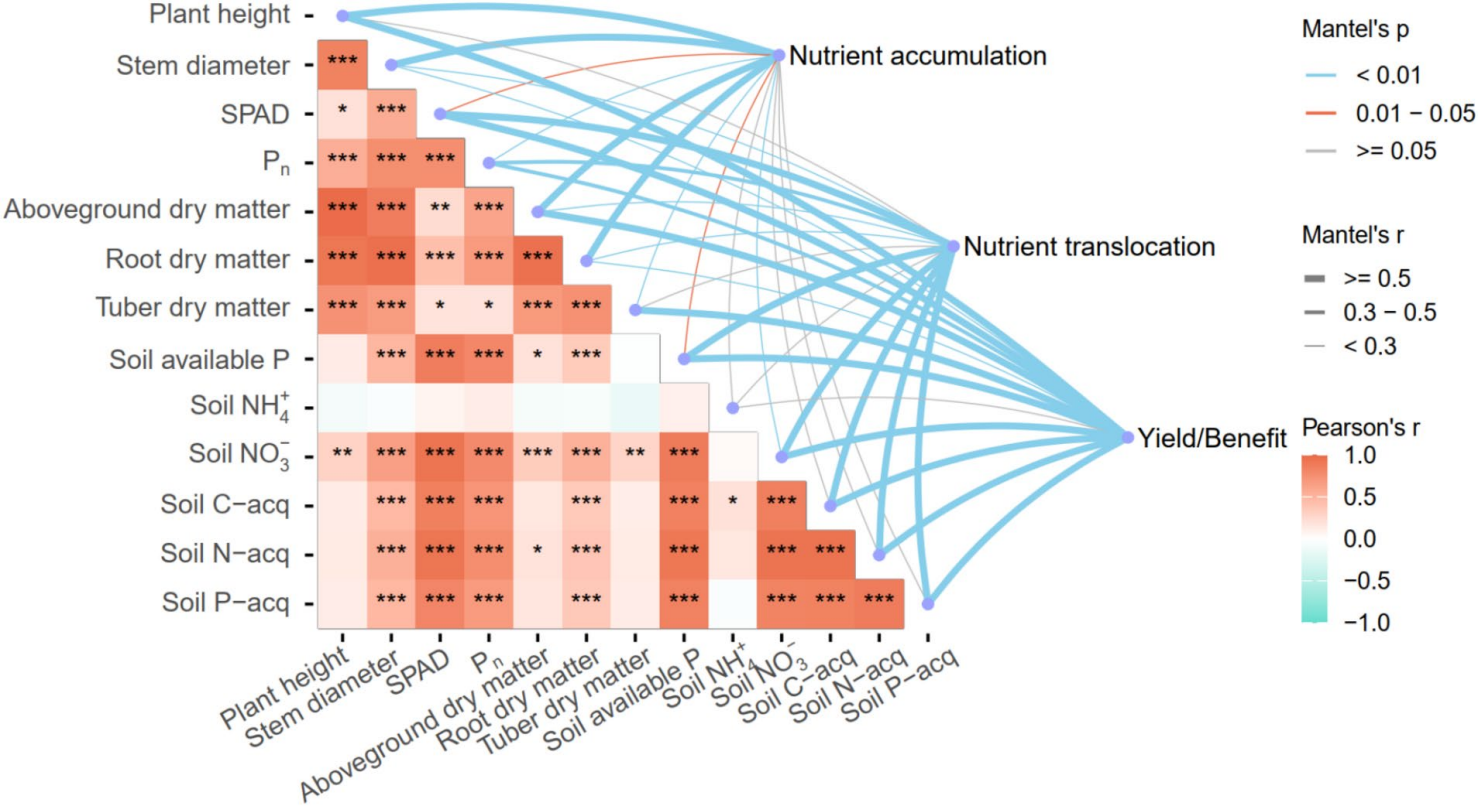
Correlation analysis between soil nutrient availability, plant growth traits, and nutrient-use efficiency under PGPR. Pearson’s correlation coefficients are shown in the lower triangle, with the significance levels indicated as *p* < 0.05, *p* < 0.01, and *p* < 0.001. The thickness and color of connecting lines represent Mantel’s *p*-values and correlation strengths between variable groups. Blue lines indicate significant positive correlations (*p* < 0.05), while red lines indicate negative correlations.

The correlation matrix plot (Figure 6) illustrates clear interaction patterns among soil and plant parameters. Nutrient accumulation exhibited strong associations with plant growth characteristics such as plant height, stem thickness, aboveground and root biomass, and showed a moderate association with the net photosynthetic rate (*P_n_*). Nutrient translocation was primarily associated with SPAD, soil available P, and soil enzyme activities. Yield formation was closely linked to soil enzyme activities, soil available P, and biomass indicators such as aboveground and tuber dry matter, as well as plant height. Furthermore, soil enzyme activities were significantly correlated with stem diameter, SPAD, *P_n_*, root dry matter, and soil available P.

## 4 Discussion

### 4.1 PGPR enhance soil nutrient availability and enzyme activities

**Figure 7:**
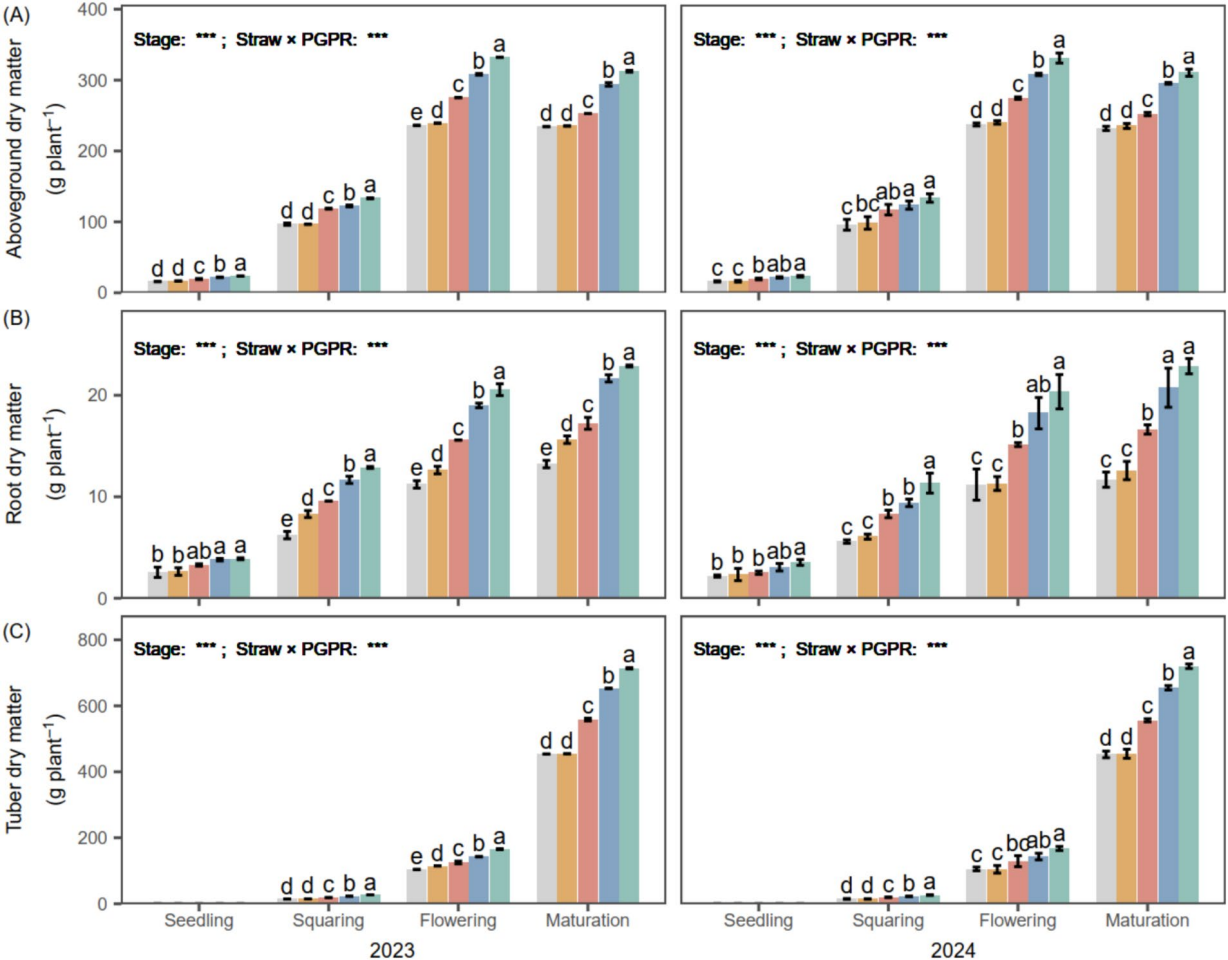
Aboveground **(A)**, root **(B)**, and tuber **(C)** dry matter accumulation of potato under different PGPR in 2023 and 2024. Different lowercase letters denote significant differences (*p* < 0.05) among seed-coating treatments at each potato growth stage. “Stage” and “Stage × PGPR” represent the main effects and their interaction in the two-way ANOVA (ns, not significant; *, **, ***, significant at increasing levels).

**Figure 8:**
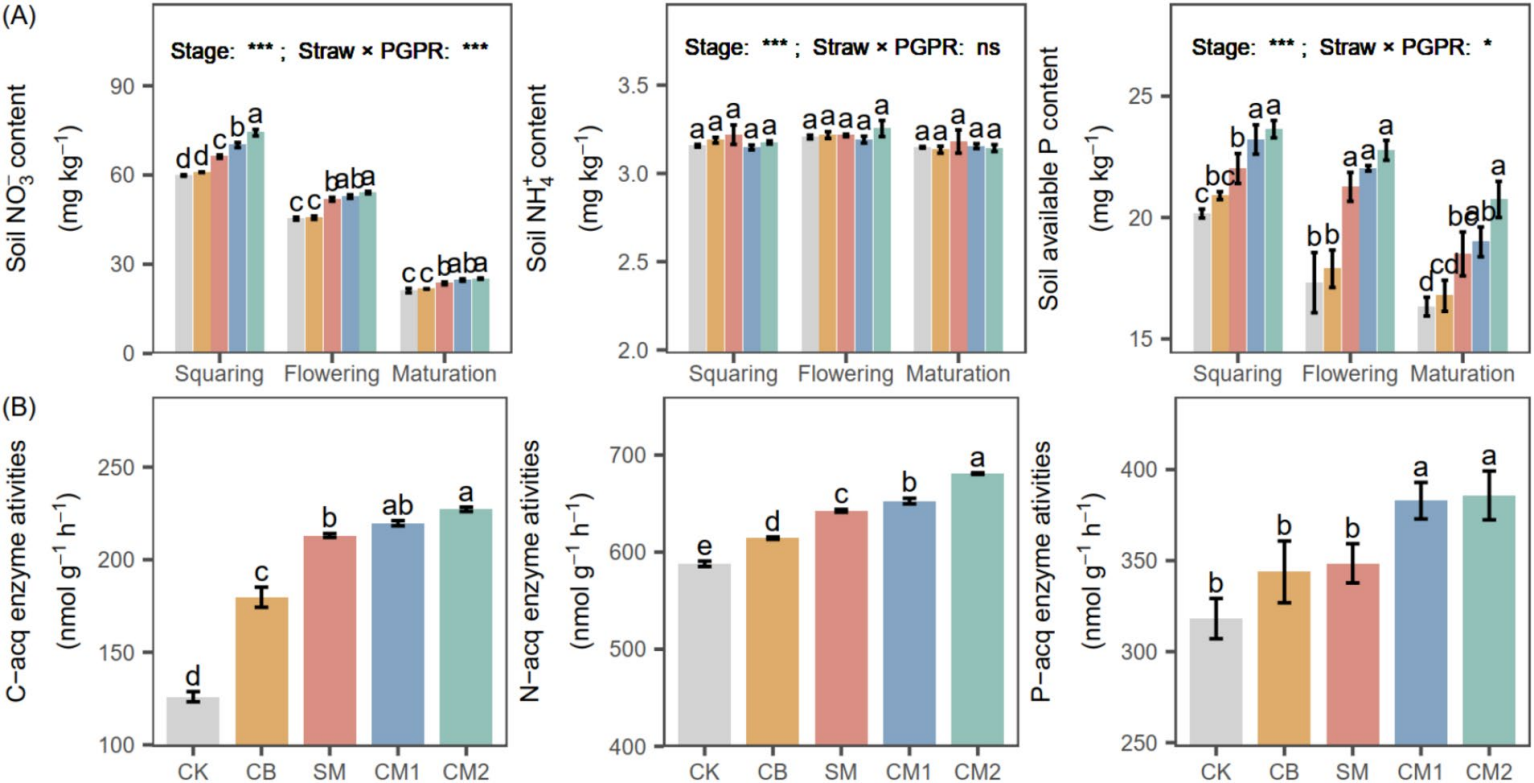
Soil nutrient contents (**A**: NO_3_–N, NH_4_^+^-N, and available P) at different growth stages and soil enzyme activities (**B**: C-, N-, and P-cycle enzyme activities) under different PGPR. Different lowercase letters denote significant differences (*p* < 0.05) among seed-coating treatments at each potato growth stage. “Stage” and “Stage × PGPR” represent the main effects and their interaction in the two-way ANOVA (ns, not significant; *, **, ***, significant at increasing levels). Samples at the harvest stage were collected at physiological maturity.

Seed-coating treatments promoted root system development (Figure 7), which increased rhizosphere enzyme activity and enhanced the secretion of organic acids capable of mobilizing insoluble phosphorus fractions, thereby improving soil nutrient availability (Khashi U Rahman et al., 2021). The chemical coating likely influenced early nutrient availability by supplying readily soluble fertilizer components directly to the rhizosphere, leading to a short-term increase in soil nutrient concentrations rather than inducing changes in soil mineral dissolution processes (Sharma and Sharma, 2025). Notably, the *Bacillus*-based seed coating (PGPR) provided continuous biological stimulation that maintained nutrient transformation throughout crop growth (Figure 4). At the flowering stage, PGPR seed coating increased soil NO₃^−^-N and available P contents by 16.29 and 17.29%, respectively, compared with the control treatments (Figure 4). This occurs because these microorganisms convert organic phosphorus compounds into available inorganic forms and release bound phosphorus by acidifying the surrounding soil environment (Lin et al., 2023), thereby increasing the level of available P in the soil. Additionally, certain PGPR strains possess nitrogen-fixing capability (Asghar et al., 2004), enabling them to transform atmospheric N₂ into plant-available ammonia (NH₃) through biological fixation (Wang et al., 2021), thereby supplying nitrogen for plant nutrition. These biological processes collectively contribute to improved nutrient cycling. The enhancement of these nutrient processes is closely related to increased soil enzyme activities (Daunoras et al., 2024). Our findings showed that the activities of enzymes involved in C, N and P cycling were markedly enhanced in PGPR-treated soils relative to the others (Figure 8). This observation is consistent with previous findings suggesting that *B. subtilis* application markedly enhances soil enzyme activities and further increases NO₃^−^N and available P contents by approximately 26.26%, thereby promoting a coordinated improvement in soil nutrient supply for crop growth (Guo et al., 2025).

### 4.2 PGPR promote potato nutrient acquisition and accelerate overall plant growth

The enhanced soil nutrient transformation and enzyme activities induced by PGPR contributed to improved agronomic traits, indicating a close linkage between soil biochemical processes and crop performance (Hu et al., 2024). In this study, PGPR markedly increased plant height and stem diameter at the flowering stage (Figure 2), which can be attributed to greater root biomass that promoted nutrient translocation to the shoots (Kramer-Walter and Laughlin, 2017). The root dry weight under PGPR seed coating was 1.62 times higher than that under the chemical coating (CB), demonstrating stronger root development. Similarly, previous studies have reported that PGPR substantially promoted potato seedling growth, primarily by promoting root biomass accumulation, which was approximately 1.7-fold greater than that in the control treatment (Won et al., 2019). Compared with the chemical coating (CB), which mainly acts through short-term chemical dissolution and disease suppression, PGPR provided continuous biological stimulation throughout the growth cycle (Sharma and Sharma, 2025). CB slightly improved early nutrient uptake and root growth, but its effect on chlorophyll maintenance and photosynthetic efficiency was limited (Figure 2). In contrast, PGPR not only enhanced root nutrient uptake capacity but also increased SPAD values and net photosynthetic rates (*P_n_*) (Gkarmiri et al., 2015), indicating improved photosynthetic efficiency and carbon assimilation. These improvements facilitated assimilate accumulation and carbon allocation to tubers, thereby increasing total biomass. In this study, tuber dry matter content under PGPR treatment was 41.88% higher than that under CB. These results collectively demonstrate that PGPR enhance potato growth by improving root biomass, photosynthetic efficiency, and assimilate partitioning. Such synergistic regulation of soil nutrient transformation and plant physiological processes ultimately contributes to greater tuber biomass and crop yield (Mun et al., 2024).

### 4.3 PGPR enhanced nitrogen and phosphorus uptake and translocation ultimately increasing potato yield

The promotion of photosynthetic activity by PGPR plays a central role in enhancing subsequent nutrient dynamics. Increased photosynthetic rate (*P_n_*) was accompanied by greater nutrient accumulation and translocation (Figure 4), suggesting that PGPR-stimulated photosynthesis strengthens carbon-nutrient interactions to promote nutrient uptake, redistribution, and yield formation (Su et al., 2024). This enhancement can be explained by the ability of PGPR to increase leaf chlorophyll content, thereby facilitating CO₂ fixation and improving photosynthetic efficiency (Khoso et al., 2024). The resulting improvement in carbon assimilation provides additional energy and carbon skeletons for nitrogen assimilation (Hu et al., 2025), which enhances nutrient translocation from source leaves to developing tubers and ultimately supports tuber bulking and yield formation. Consistently, PGPR significantly enhanced potato N and P accumulation (Figure 7) and further increased the N and P translocation rates by an average of 17.13 and 50.48%, respectively, compared with CK and CB over the 2 years (Figure 3). Comparable reallocations of photosynthates from source to storage organs have been documented (Zhou et al., 2018; Li et al., 2020; Wu et al., 2021), corroborating the inference that PGPR-enhanced photosynthesis accelerates carbon flow and nutrient allocation toward developing tubers. Similar physiological responses have been observed in potato and other C₃ crops, where PGPR-induced enhancement of photosynthetic efficiency significantly contributed to nitrogen assimilation and biomass allocation (Singh et al., 2019; Zagorščak et al., 2025).

Besides the stimulation of photosynthesis, the higher nutrient accumulation under PGPR could also be attributed to enhanced root nutrient acquisition and microbially mediated improvement in soil N and P availability, jointly contributing to greater nutrient uptake and utilization efficiency (Su et al., 2024). This inference is supported by the increases in soil available N and P contents, root biomass, and enzyme activities observed under PGPR (Figures 3, 7), indicating that both nutrient supply and root absorption capacity were improved. Such synergistic regulation of above- and below-ground processes strengthens the coupling between carbon assimilation and nutrient uptake, thereby enhancing nutrient acquisition, translocation, and utilization, which collectively contribute to improved potato yield (Tang et al., 2025; Zhang et al., 2025). Furthermore, the significant positive correlations between photosynthetic rate (*P_n_*) and plant N and P accumulation (Figure 4) further support the tight linkage between enhanced photosynthetic performance and improved nutrient utilization efficiency.

## 5 Conclusion

In this two-year field experiment, potato seed coating with PGPR improved soil nutrient dynamics, plant physiological performance, and potato yield. PGPR led to pronounced increases in soil available nutrient levels and the activities of enzymes associated with C, N, and P cycling, thereby stimulating nutrient mineralization and turnover within the rhizosphere. Enhanced soil nutrient availability and root biomass promoted nutrient acquisition and translocation, thereby increasing N and P accumulation in both aboveground organs and tubers. PGPR also improved leaf chlorophyll content and photosynthetic rate, strengthening carbon-nutrient coupling and source-sink coordination. Consequently, PGPR treatments-particularly the composite inoculant *Bacillus subtilis* + *Bacillus licheniformis* (CM2)-increased the N and P translocation, and improved yield relative to the untreated control. PGPR optimize soil biochemical and plant physiological processes, enhancing nutrient uptake and utilization, while composite Bacillus-based seed coatings provide a sustainable strategy to improve soil fertility and potato productivity.

## Data Availability

The original contributions presented in the study are included in the article/supplementary material, further inquiries can be directed to the corresponding authors.
